# Genotype-Phenotype Correlation Reanalysis in 83 Chinese Cases with *OCRL* Mutations

**DOI:** 10.1155/2022/1473260

**Published:** 2022-07-19

**Authors:** Lingxia Zhang, Shugang Wang, Ruoque Mao, Haidong Fu, Jingjing Wang, Huijun Shen, Zhihong Lu, Junyi Chen, Yu Bao, Chunyue Feng, En Yin Lai, Qing Ye, Jianhua Mao

**Affiliations:** ^1^Department of Nephrology, The Children's Hospital of Zhejiang University School of Medicine, Hangzhou, China; ^2^Chigene (Beijing) Translational Medical Research Center, E2 Biomedical Park, No. 88 Kechuang Sixth Ave, Yizhuang, Beijing, China; ^3^Department of Physiology, Zhejiang University School of Medicine, Hangzhou, China

## Abstract

**Background:**

Both Lowe syndrome and Dent-2 disease are caused by variants in the *OCRL* gene. However, the reason why patients with similar *OCRL* gene mutations presented with different phenotypes remains uncertain.

**Methods:**

Children with hemizygous pathogenic or likely pathogenic variants in *OCRL* were compiled from published and unpublished consecutive cases from China. Furthermore, a Chi-square test was employed to analyze the correlation of the location and types of mutations on the phenotype of children with Lowe syndrome or Dent-2 disease.

**Results:**

Among the total 83 patients, 70.8% (34/48) cases of Lowe syndrome presented with truncating mutations, while only 31.4% (11/35) cases of Dent-2 disease presented with truncating mutation (Χ^2^ = 12.662; *P* < 0.001). Meanwhile, the majority of mutations in Dent-2 disease are located in Exon 2–12 (21/35, 60.0%), while the majority of mutations in Lowe syndrome are located in Exon 13–23 (39/48, 81.3%; Χ^2^ = 14.922; *P* < 0.001).

**Conclusions:**

Truncating mutations of the *OCRL* gene were more common in patients with Lowe syndrome than in Dent-2 disease, while mutation is more likely located at exon 2–12 in Dent-2 disease than that in Lowe syndrome. The type and location of mutation are important indicators for the phenotypes in patients with *OCRL* mutation. This is a large cohort study analyzing the genotype-phenotype correlation in patients with Lowe syndrome and Dent-2 disease in China. Our data may improve the interpretation of new *OCRL* variants and genetic counseling. Furthermore, a large international study would be necessary to illustrate the genotype-phenotype correlation in patients with *OCRL* mutations.

## 1. Background

Lowe syndrome, or oculo-cerebro-renal syndrome, is a rare X-linked multisystemic disorder characterized by the triad of congenital cataracts, intellectual disability, and proximal renal tubular dysfunction with slowly progressive renal failure [1]. Lowe syndrome is caused by variants in the *OCRL* gene on chromosome Xq25-26. In the meantime, mutations in the *OCRL* also can lead to a type 2 Dent disease (Dent-2 disease) that can present as isolated proximal renal tubulopathy, characterized by low-molecular-weight proteinuria (LMWP), hypercalciuria, and progressive renal insufficiency in the absence of extrarenal pathologies [2]. Except for rickets noted in some patients, no extrarenal manifestations of the disease have been reported in Dent-2 disease.

To date, a hypothesis of phenotype-genotype correlation exists between Dent-2 disease and Lowe syndrome [3]. Disease-causing mutations occur throughout the *OCRL* gene in patients with Lowe syndrome, but mainly in exons 9–15, which encode the catalytic domain of the protein. In the meantime, disease-causing mutations from patients with Dent-2 disease occur mainly in exons 1–8, which mainly encode the PH domain of the protein. This continuum was not only observed between patients harboring different *OCRL* mutations but also occurred between patients harboring the same mutation [4]. Understanding how mutations in *OCRL* give rise to two clinical entities with differing extrarenal manifestations represents an opportunity to identify molecular pathways that could be targeted to develop treatments for these conditions [4].

In the present study, 48 consecutive Chinese children with Lowe syndrome and 35 with Dent-2 disease from published or new data were collected and reanalyzed to further understand the phenotype-genotype correlation of the *OCRL* gene.

## 2. Methods

### 2.1. The Aim, Design, and Setting of the Study

Both Lowe syndrome and Dent-2 disease are caused by variants in the OCRL gene. However, the reason why patients with similar OCRL gene mutations presented with different phenotypes remains uncertain. The aim of the present study is to analyze the correlation between the genotype of *OCRL* gene mutations and the phenotype of children with Lowe syndrome or Dent-2 disease.

### 2.2. The Characteristics of Participants

Between January 2010 and July 2020, 5 probands with a clinical diagnosis of Lowe syndrome and 4 probands with a clinical diagnosis of Dent-2 disease were recruited at the Children's Hospital of Zhejiang University School of Medicine.

Appropriate informed consent was obtained from all patients and their families. They were recruited according to the classical criteria for Lowe syndrome or Dent disease, respectively.

The patients who presented with full oculo (congenital cataract, and congenital glaucoma), cerebro (hypotonia, developmental delay, and mental retardation), and renal symptoms were diagnosed with Lowe syndrome, while the patients who presented with isolated renal tubulopathy were identified with Dent-2 disease.

The pediatric patients who met at least two of the following criteria for Dent-2 disease and detected *OCRL* gene mutations were eventually included in this cohort [5, 6]. (1) LMWP: early renal injury index indicates that the low-molecular-weight protein in urine is at least five times higher and is mainly LMWP. The low-molecular-weight proteins used for monitoring mainly include retinol-binding protein and *α*1-MG. *β*2-Microglobulin or urine protein electrophoresis suggests that low-molecular-weight protein accounts for more than 50%. (2) Hypercalciuria: 24-hour urinary calcium > 4 mg/kg (> 1 mmol/kg) or increased random urinary calcium/urine creatinine ratio (random urinary calcium/urine creatinine ratios vary strongly with age; specific criteria refer to a previous report) [7]; (3) one of the following conditions: microscopic hematuria, kidney stones, nephrocalcinosis, hypophosphatemia, or renal insufficiency.

The exclusion criteria included the following: (a) the families who refused the genetic test or participant registration and those who (b) failed the quality control (mean depth coverage of less than × 20 or target coverage region < 90%).

The clinical features and family history of 8 probands in this cohort are presented in Tables 1 and 2.

Estimated glomerular filtration rates (eGFR) were calculated by the Schwartz equation [8] which was applied for patients <18 years. One hundred healthy children were selected as control subjects.

The study protocol was approved by the Ethics Committee of the Children's Hospital of Zhejiang University School of Medicine. All study participants or their guardians provided written informed consent before enrollment.

Furthermore, we conducted a systematic search in 3 English databases and 4 Chinese databases: MEDLINE, Cochrane Library, EMBASE database, CNKI, Wanfang database, the Chinese Scientific Journal database, and the Chinese biomedical literature service system. Studies were identified using keyword search terms: “Lowe syndrome,” ”oculo-cerebro-renal syndrome” and “Dent disease.” The search was limited from January 2010 to July 2020 among all the above databases.

Studies were eligible for inclusion if (i) published in Chinese or English with the cases from China. (ii) Study population: children with diagnosis under 18 years. (iii) Genetic sequencing with *OCRL* mutations.

### 2.3. Mutation Analysis (As Mentioned before)

#### 2.3.1. DNA Extraction

Genomic DNA was extracted from 5 mL of the peripheral blood of patients by a QIAamp Blood DNA Mini Kit (Qiagen, Milano, Italy) according to the manufacturer's instructions [6, 9]. DNA concentrations were measured by a NanoDrop spectrophotometer (Thermo Scientific, Waltham, MA, USA). DNA samples were then stored at −20°C until use.

### 2.4. Whole-Exome Sequencing

Exome sequencing was performed in two pools to optimize the results. Samples were pooled based on the clinical features of the patients. An array capture was used to enrich the relevant human genes (SeqCap EZ Human Exome Library v2.0, Roche®, Basel, Switzerland), and these genes were sequenced on the Illumina HiSeq 2000 platform (Illumina, Inc, USA).

### 2.5. Data Filtering

The following initial steps were performed to prioritize the high-quality variants: (i) variants within intergenic, intronic, and untranslated regions (UTRs) and synonymous mutations were excluded from downstream analysis; (ii) variants with a quality score < 20 were excluded; (iii) only conservation scores (phyloP) >3 were considered upon a comparison of humans and 43 other vertebrates. After the initial selection, the remaining genes were filtered by function. PolyPhen-2 software was used to predict the possible impact of variants (http://genetics.bwh.harvard.edu/pph2/). The final set of selected variants was visually inspected using the Integrative Genomics Viewer. Thirteen polymorphic variants previously described in public databases were investigated and compared with the variations found in the current exome. The selected mutations investigated in this study were not found in previous exome sequences (http://evs.gs.washington.edu/EVS/).

### 2.6. Sanger Sequencing Validation

To confirm the NGS data, Sanger sequencing was employed. DNA from all diagnosed children and their parents were subjected to a polymerase chain reaction (PCR), and polyacrylamide gel electrophoresis was used to determine the size of the amplification products. Products were purified using the QIAquick PCR Purification Kit (Qiagen, Milano, Italy) and sequenced with both forward and reverse primers using the ABI BigDye Terminator Cycle Sequencing Kit v. 3.1 on an ABIPRISM 3730XL Genetic Analyzer (Applied Biosystems, Foster City, CA, USA). The results were aligned with reference sequences, and mutations were identified using Sequencher DNA Sequence Analysis Software (http://www.genecodes.com). All primers were designed using the online tool Primer3 (http://sourceforge.net/projects/primer3/).

### 2.7. Mapping and Protein Structure Prediction

Protein and DNA sequence alignments were performed by ClustalW (http://www.geno-me.jp/tools-bin/clustalw) and MultAlin (http://multalin.toulouse.inra.fr/multalin/), respectively. The predicted effects of amino acid substitutions on the biological function of the protein were evaluated using both PolyPhen-2 and Provean software (http://genetics.bwh.harvard.edu/pph2/ and http://provean.jcvi.org, respectively).

## 3. Results

### 3.1. Patients with Lowe Syndrome or Dent-2 Disease

Between January 2010 and July 2020, 9 consecutive patients (5 with Lowe syndrome and 4 with Dent-2 disease) were recruited and sequenced at the Children's Hospital of Zhejiang University School of Medicine.

Furthermore, 43 consecutive patients with Lowe syndrome and 31 consecutive patients with Dent-2 disease were collected from published data from January 2010 to July 2020.

**Figure 1:**
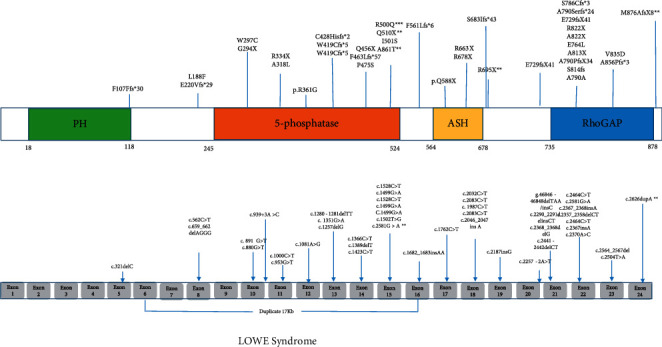
Exon structure of the *OCRL* gene with geometric shapes indicating relative positions of different types of mutations in Lowe syndrome.

**Figure 2:**
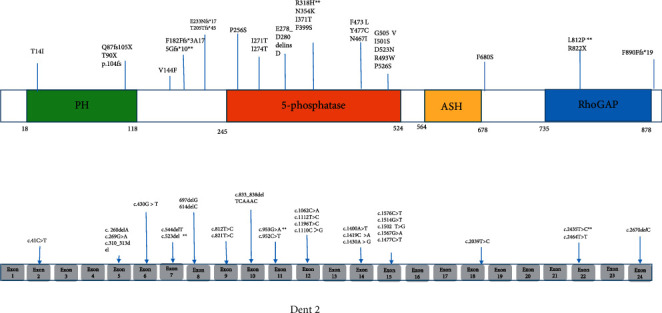
Exon structure of the OCRL gene with geometric shapes indicating relative positions of different types of mutations in Dent-2 disease. Differences of mutation type and mutation location between Lowe syndrome and Dent-2 disease.

Therefore, the clinical manifestation and mutation profile of the *OCRL* gene from total of 48 patients with Lowe syndrome and 35 patients with Dent-2 disease were summarized in Table 3 and Figure 1 (Lowe syndrome), and Table 1 and Figure 2 (Dent-2 disease), respectively.

To compare the difference in mutation type between Lowe syndrome and Dent-2 disease, all types of mutations were summarized as truncating mutation (nonsense, splicing defect, and incomplete insertion or deletion resulting frameshift and truncated protein) and nontruncating mutation (missense mutation, small in-frame insertion or deletion). The results of mutation type are summarized in Table 2 from patients with Lowe syndrome and Dent-2 disease.

Compared with the results from the literature [1, 2], the results of mutation location (Exon 1–7 for Dent-2 disease vs. Exon 8–23 for Lowe syndrome) are summarized in Table 4 from patients with Lowe syndrome and Dent-2 disease.

Furthermore, the results of mutation location (exon 2–12 for Dent-2 disease vs. exon 13–23 for Lowe syndrome) are summarized in Table 5 from patients with Lowe syndrome and Dent-2 disease.

## 4. Discussion

Mutations affecting the *OCRL* gene were primarily associated with Lowe syndrome, and subsequently with Dent-2 disease. More than 140 pathogenic mutations in *OCRL* have been described so far and reported throughout the entire gene from exon 2 to exon 23. Their phenotype is influenced significantly by the genotype (the mutation type and location of the disease-causing gene and related surrogate gene).

In the present study, 48 series of cases of Lowe syndrome and 35 cases of Dent-2 disease were recruited. Among them, 34 in 48 cases of Lowe syndrome presented with truncating mutations, while 11 in 35 cases of Dent-2 disease presented with truncating mutation, demonstrating that truncating mutations of *OCRL* gene were more likely seen in patients with Lowe syndrome than Dent-2 disease.

In 2009, from 6 cases of Dent-2 disease reported by Shrimpton et al. [2], all missense mutations fall in the phosphatidylinositol phosphate 5-phosphatase domain of the *OCRL* protein, while all the other mutations, nonsense, and frameshift fall in the first 7 exons of the gene. They concluded that this distribution suggests that two different classes of mutations underlie the two diseases (Lowe syndrome and Dent-2 disease).

In 2011, from a large cohort study recruited 130 Lowe families and 6 Dent-2 disease cases by Hichri et al. [1], the specific mapping of the frameshift and nonsense mutations, exclusively identified in exons 1–7 and exons 8–23, respectively, for Dent-2 disease and Lowe syndrome together with the possible use of alternative initiation codons might be related to their clinical expression, that is, Lowe syndrome or Dent-2 disease.

Recently, a large international study [3] of *OCRL* variants widened the range of exons leading to Dent-2 disease phenotype demonstrating that mutations in Lowe syndrome are located among exons 8 and 24, while exons 4–15 are affected in Dent-2 disease. There are also reported cases in which *OCRL* mutations affecting exons at the 3′ side of exon 15 led to the Dent-2 disease phenotype [10].

To further demonstrate the correlation between mutation location and phenotype of Lowe syndrome or Dent-2 disease, Chi-square testing was employed to analyze the difference in mutation location between Lowe syndrome and Dent-2 disease in the present study, and the results demonstrated the majority of mutations in Dent-2 disease are located in exon 2–12 (21/35, 60.0%), while the majority of mutations in Lowe syndrome are located in exon 13–23 (39/48, 81.3%, Χ^2^ = 14.922, *P* < 0.001).

Alternatively, if reconsideration of the “cutoff” value of mutation location to exon 2–7 for Dent-2 disease, and exon 8–23 for Lowe syndrome, as suggested by Hichri et al. [1] and Shrimpton et al. [2], then the results demonstrated that 8 in 35 cases with Dent-2 disease are located in exon 2–7 (22.9%), while 47/48 cases with Lowe syndrome are located in exon 8–23 (97.9%), and a significant difference was found between two groups (Χ^2^ = 9.035, *P*=0.003).

Furthermore, after reanalysis of the results, the distribution of the mutations in Figures 1 and 2 in the present study suggests that (1) pathogenic variants in exon 2–9 are clearly more associated with Dent-2 disease; (2) pathogenic variants in exon 16–23 preferentially cause Lowe syndrome; (3) variants in exon 10–15 can cause one or the other condition in a similar ratio.

*OCRL* is a multidomain protein of 110kDa. In addition to its 5-phosphatase catalytic domain, it contains a pleckstrin homology (PH) domain, an ASPM, SPD-2, Hydin (ASH) domain, characteristic of proteins that localize to centrosomes and primary cilia, and a RhoGAP-like domain, which mediates the interaction of *OCRL* with Cdc42 and Rac1. According to the results of the present study and others, it is speculated that mutations in the N-terminal domain of *OCRL*, where most Dent-2 disease mutations are located, would allow the expression of splicing variants that retain some biological activity. For this reason, this apparent difference between the two diseases might be explained by the partial preservation of other noncatalytic functions of the protein in patients with Dent-2 disease or by the presence of other modifier genes.

## 5. Conclusions

This is a large cohort study to reanalyze the genotype-phenotype correlation in patients with Lowe syndrome and Dent-2 disease in China. The results demonstrated truncating mutations of the *OCRL* gene were mostly seen in patients with Lowe syndrome than in Dent-2 disease, while mutation of the *OCRL* gene is more likely located at exon 2–12 in Dent-2 disease than that in Lowe syndrome. Our data may improve the interpretation of new *OCRL* variants and genetic counseling.

## Figures and Tables

**Table 1:** Mutations of the *OCRL* gene in patients with Dent-2 disease.

Patient ID	Age of onset	Exon	Nucleotide change	Protein change	Result	Segregation	LMWP	Hypercalciuria	Nephrocalcinosis or nephrolithiasis
2014-01 [35]	6Y	5	c.260delA	p.Q87fs105X	Frameshift deletion	M	√	√	none
2016-02 [36]	3.4Y	22	c.2435T > C	p.L812P	Missense	Unknown	√	√	none
2016-03 [37]	2Y	15	c.1576C > T	p.P526S	Missense	Unknown	√	√	Nephrolithiasis
2016-04 [38]	4Y	22	c.2435T > C	p.L812P	Missense	M	√	√	None
2016-05 [39]	4.4Y	10	c.833_838delTCAAAC	p.E278_D280delinsD	Nonframeshift	M	√	None	None
2016-06 [39]	2.5Y	10	c.833_838delTCAAAC	p.E278_D280delinsD	Nonframeshift	M	√	None	None
2016-07 [39]	1Y	7	c.523del	p.A175Gfs^*∗*^10	Frameshift deletion	M	√	None	None
2016-08 [39]	0.7Y	7	c.523del	p.A175Gfs^*∗*^10	Frameshift deletion	M	√	None	None
2018-09 [40]	9Y	12	c.1062C > A	p.N354K	Missense	De novo	√	√	None
2018-10 [41]	4Y	9	c.812T > C	p.I271T	Missense	De novo	√	√	Nephrocalcinosis
2018-11 [42]	9Y3M	9	c.821T > C	p.I274T	Missense	M	√	√	None
2018-12 [42]	11Y8M	12	c.1112T > C	p.I371T	Missense	M	√	None	None
2018-13 [42]	7Y10M	12	c.1196T > C	p.F399S	Missense	M	√	√	None
2019-14 [43]	3Y	11	c.953G > A	p.R318H	Missense	M	√	√	None
2019-15 [43]	3Y	15	c.1477C > T	p.R493W	Missense	M	√	√	None
2020-16 [6]	2.2Y	14	c.1419C > A	p.F473L	Missense	M	√	√	None
2020-17 [6]	3.33Y	14	c.1430A > G	p.Y477C	Missense	M	√	√	None
2020-18 [6]	10Y	24	c.2670delC	p.F890Ffs^*∗*^19	Frameshift	M	√	√	Nephrolithiasis
2020-19 [6]	5Y	15	c.1514G > T	p.G505V	Missense	M	√	√	None
2020-20 [6]	1.75Y	8	c.697delG	p.E233Nfs^*∗*^17	Frameshift	M	√	None	Nephrocalcinosis
2020-21 [6]	2Y	8	c.614delC	p.T205Tfs^*∗*^45	Frameshift	M	√	√	Nephrocalcinosis
2020-22 [6]	2Y	6	c.430G > T	p.V144F	Missense	M	√	√	None
2020-23 [6]	0.92Y	15	c.1502T > G	p.I501S	Missense	M	√	√	Nephrocalcinosis
2020-24 [6]	2.08Y	22	c.2464C > T	p.R822X	Nonsense	M	√	√	None
2019-25 [44]	5.3Y	15	c.1567G > A	p.D523N	Missense	M	√	√	Nephrolithiasis
2019-26 [44]	3Y	7	c.544delT	p.F182Ffs^*∗*^3	Frameshift deletion	M	√	√	Nephrolithiasis
2019-27 [44]	3.8Y	5	c.310_313del	p.104fs	Missense	M	√	√	Nephrocalcinosis
2020-28 [45]	3.9Y	22	c.2435T > C	p.L812P	Missense	M	√	√	None
2020-29 [45]	7.2Y	12	c.1110C > G	p.C370T	Missense	M	√	√	None
2020-30 [45]	3.8Y	5	c.269G > A	p.T90X	Nonsense	M	√	√	None
2020-31 [46]	9Y	14	c.1400A > T	p.N467I	Missense	M	√	√	None
2020-32^*∗*^	10Y	*CLCN5*-E6	c.638C > T	p.P213L	Missense	M	√	√	Nephrocalcinosis, hematuria
		*OCRL*-E2	c.41C > T	p.T14I	Missense	M			
2020-33^*∗*^	3Y	11	c.953G > A	p.R318H	Missense	De novo	√	√	Nephrolithiasis
2020-34^*∗*^	1Y1M	18	c.2039T > C	p.F680S	Missense	M	√	√	Nephrocalcinosis
2020-35^*∗*^	2Y	11	c.952C > T	p.R318C	Missense	M	√	√	Hematuria

LMWP, low-molecular-weight-proteinuria; ^*∗*^Case from the present study; Segregation: M: the proband's mother carried the mutation.

**Table 2:** Mutations of the *OCRL* gene in patients with Lowe syndrome.

Patient ID	Age of onset	Exon	Nucleotide change	Protein change	Result	Segregation	Ocular symptoms	Neurological symptoms	Renal involvements
2011-01 [12]	5Y	18	c.2032C > T	p.R678X	Nonsense	M	CC	DD, MR, epilepsy	LMWP
2011-02 [13]	Fetus	18	c.2046_2047 ins A	p.S683Ifs^*∗*^43	Frameshift	M	Unknown	Unknown	Unknown
2012-03 [14]	11M	15	c.1528C > T	p.Q510X	Nonsense	De novo	CC, CG	DD, MR	FS, PT
2012-04 [15]	9Y	10	c.880G > T	p.G294X	Nonsense	M	cc	DD	LMWP, rickets
2012-05 [15]	26Y	24	c.2626dupA	p.M876AfsX8	Frameshift insertion	M	CC	HY, DD	LMWP, ALP
2012-06 [15]	32Y	24	c.2626dupA	p.M876AfsX8	Frameshift insertion	M	CC	Hy, MR, DD	LMWP
2014-07 [16]	9M	15	c.1499G > A	p.R500Q	Missense	M	CC	DD, MR	LMWP
2015-08 [17]	2Y1M	8	c.562C > T	p.L188F	Missense	M	CC	DD, MR	PT, LMWP
		22	c.2464C > T	p.R822X	Nonsense	M			
2015-09 [18]	2Y9M	Intron 20	g.46846–46848delTAA/insC	Splicing defect	Unknown	CC	DD, MR	Rickets, LMWP	
2015-10 [18]	1Y3M	5	c.321delC	p.F107Ffs^*∗*^30	Frameshift deletion	Unknown	CC	MR	PT, LMWP
2015-11 [19]	3Y	22	c.2367insA	p. A813X	Nonsense	M	CC	DD,Hy	Rickets, LMWP
2016-12 [20]	0.9Y	15	c.1528C > T	p.Q510X	Nonsense	De novo	CC	DD, MR	Rickets, PT, LMWP
2016-13 [20]	5Y	19	c.2187insG	p.E729fsX41	Insertion	M	CC	DD, MR	Rickets, LMWP
2016-14 [20]	5Y	14	c.1366C > T	p.Q456X	Nonsense	De novo	CC	DD, MR	Rickets, LMWP
2016-15 [20]	0.2Y	15	c.1499G > A	p.R500Q	Missense	unknown	CC	DD, MR	LMWP
2016-16 [20]	2Y	22	c.2581G > A	p.del exon 22	Splicing	De novo	CC	DD, MR	LMWP
2016-17 [21]	10M	13	c.1280–1281delTT	p.C428Hisfs^*∗*^2	Frameshift deletion	De novo	CC	DD	LMWP
2016-18 [22]	3Y	18	c.2083C > T	p.R695X	Nonsense	M	CC	DD, MR	Rickets, LMWP
2016-19 [22]	4M	21	c.2441–2442delCT	p. S814fs	Frameshift deletion	M	CC	DD, Hy	AA, LMWP
2017-20 [23]	Fetus		Xq25-26.1del	633kb	Full length deletion	M	CC	Cerebral dysplasia	Unknown
2017-21 [24]	4Y	11	c.953G > T	p.A318L	Missense	De novo	CC	DD	LMWP
2018-22 [25]	2Y5M	15	C.1499G > A	p.R500Q	Missense	M	CC	DD, MR	LMWP
2019-23 [26]	2Y8M	11	c.1000C > T	p.R334Stop	Stop code	M	CC	DD, MR	Rickets, LMWP
2019-24 [26]	2Y5M	18	c.2083C > T	p.R695Stop	Stop code	M	CC	DD, MR	Rickets, LMWP
2019-25 [27]	11M	14	c.1389delT	p.F463Lfs^*∗*^57	Frameshift deletion	M	CC	DD, MR	Rickets, LMWP
2019-26 [28]	6M		Xq25-26.1del	249kb	Full length deletion	Unknown	CC	DD, MR	LMWP
2019-27 [29]	2Y	14	c.1423C > T	p.P475S	Missense	M	CC	Hy, MR, DD	LMWP
2019-28 [29]	2Y	22	c.2464C > T	p. A822X	Nonsense	M	CC	Hy, MR, DD	LMWP
2019-29 [29]	11M	15	c.1502T > G	p. I501S	Missense	M	CC	Hy, MR, DD	LMWP
2019-30 [30]	14Y	21	c.2290_2291delinsCT	p.E764L	Missense	De novo	CC	DD	
2019-31 [30]	9Y	21	c.2581G > A	p.A861T	Missense	M	CC	DD	
2019-32 [30]	5Y	21	c.2581G > A	p.A861T	Missense	M	CC	DD	LMWP
2019-33 [6]	6Y	21	c.2368_2368delG	p.A790PfsX34	Frameshift deletion	M	CC	DD	LMWP
2019-34 [31]	unknown	IVS20	c.2257-2A > T		Splicing	Unknown	Unknown	Unknown	PT, LMWP
2019-35 [31]	unknown	8	c.659_662delAGGG	p.E220Vfs^*∗*^29	Frameshift	Unknown	Unknown	Unknown	PT, LMWP
2020-36 [32]	1Y6M	5–16	Duplicate	17.9kb		M	CC	DD, MR	LMWP
2020-37 [33]	2.7Y	22	c. 2367_2368insA	p. A790Serfs^*∗*^24	Frameshift insertion	M	CC	DD, MR, Hy	Rickets, PT, LMWP
2020-38 [33]	7.5Y	10	c. 891G > T	p.W297C	Missense	M	Mild CC	Mild MR	Rickets, LMWP
2020-39 [33]	1.3Y	13	c. 1351G > A	p. D451A	Missense	Unknown	CC, CG	DD, MR, Hy	Rickets, PT, LMWP
2020-40 [33]	1.8Y	18	c. 1987C > T	p. R663X	Nonsense	M	CC	DD, MR, Hy	Rickets, LMWP
2020-41 [33]	0.7Y	23	c. 2564_2567del	p. A856Pfs^*∗*^3	Frameshift deletion	M	CC	DD, Hy	LMWP
2020-42 [33]	0.7Y	16	c. 1682_1683insAA	p. F561Lfs^*∗*^6	Frameshift insertion	M	CC, CG	DD, Hy	Rickets, LMWP
2020-43 [34]	9Y	IVS10	c.939+3A > C		Splicing	M	CC	DD,MR	PT, LMWP
2020-44^*∗*^	1Y10M	23	c.2504T > A	p.V835D	Missense	M	CC	DD, MR	LMWP
2020-45^*∗*^	11Y	22	c.2357_2358delCT	p.S786Cfs^*∗*^3	Frameshift deletion	M	CC	DD, MR	LMWP
2020-46^*∗*^	1M9D	13	c.1257delG	p.W419Cfs^*∗*^5	Frameshift deletion	M	CC	DD, Hy	PT, LMWP
2020-47^*∗*^	5M2D	17	c.1762C > T	p.Q588X	Nonsense	M	CC	DD, Hy, MR	PT, LMWP
2020-48^*∗*^	9Y	12	c.1081A > G	p.R361G	Missense	M	CC	MR	LMWP, hypercalciuria

CC, congenital cataract; CG, congenital glaucoma; Hy, hypotonia; DD, developmental delay; MR, mental retardation; FS, Fanconi syndrome; PT, proximal tubulopathy; RF, renal failure; AA, aminoaciduria; LMWP, low-molecular-weight proteinuria. ^*∗*^Case from the present study. Segregation: M: the proband's mother carried the mutation.

**Table 3:** The results of mutation type from Lowe syndrome and Dent-2 disease.

	Lowe syndrome (*n* = 48)	Dent-2 disease (*n* = 35)
Truncating mutation	34	11
Nontruncating mutation	14	24

For Chi testing, Χ^2^ = 12.662, *P* < 0.001.

**Table 4:** Different mutation locations from Lowe syndrome and Dent-2 disease.

	Lowe syndrome (*n* = 48)	Dent-2 disease (*n* = 35)
Exon 2–7 [1, 2]	1	8
Exon 8–23 [1, 2]	47	27

For chi testing, Χ^2^ = 9.035, *P*=0.003.

**Table 5:** Different mutation locations from Lowe syndrome and Dent-2 disease.

	Lowe syndrome (*n* = 48)	Dent-2 disease (*n* = 35)
Exon 2–12	9	21
Exon 13–23	39	14

For chi testing, Χ^2^ = 14.922, *P* < 0.001.

## Data Availability

The full protein sequence of OCRL has been submitted to SWISS-MODEL (http://swissmodel.expasy.org/) servers.
